# Multiscalar electrical spiking in *Schizophyllum commune*

**DOI:** 10.1038/s41598-023-40163-z

**Published:** 2023-08-07

**Authors:** Andrew Adamatzky, Ella Schunselaar, Han A. B. Wösten, Phil Ayres

**Affiliations:** 1 Unconventional Computing Lab, UWE, Bristol, UK; 2 Microbiology, Department of Biology, Utrecht University, Utrecht, The Netherlands; 3 The Centre for Information Technology and Architecture, Royal Danish Academy, Copenhagen, Denmark

**Keywords:** Biophysics, Computational biology and bioinformatics, Mathematics and computing

## Abstract

Growing colonies of the split-gill fungus *Schizophyllum commune* show action potential-like spikes of extracellular electrical potential. We analysed several days of electrical activity recording of the fungus and discovered three families of oscillatory patterns. Very slow activity at a scale of hours, slow activity at a scale of 10 min and very fast activity at scale of half-minute. We simulated the spiking behaviour using FitzHugh–Nagume model, uncovered mechanisms of spike shaping. We speculated that spikes of electrical potential might be associated with transportation of nutrients and metabolites.

## Introduction

Organisms generate electromagnetic fields and employ the fields to obtain and fuse information about their environment, to establish communication between components of their bodies and to control the shape of their bodies^1–10^. From an information processing point of view, one from the most interesting phenomena of bio-electricity is neural spiking. Spikes of electrical potential are the most well-known attributes of neurons and are attributed to their learning and decision making^11–13^. Not only neurons, but most living substrates can produce spikes of electrical potential. These include Protozoa^14–16^, Hyrdoroza^17^, slime moulds^18,19^ and plants^20–22^.

Action potential-like spiking activity in fungi was first documented in 1976^23^, further confirmed in 1995^24^ and techniques for recording the electrical activity in fruiting bodies and colonised substrates was identified in 2018^25^. While trying to uncover mechanisms of integrative electrical communication in fungi, we recorded and analysed electrical activity of oyster fungi *Pleurotus djamor*^25^, bracket fungi *Ganoderma resinaceum*^26^, ghost fungi (*Omphalotus nidiformis*), Enoki fungi (*Flammulina velutipes*), split gill fungi (*Schizophyllum commune*) and caterpillar fungi (*Cordyceps militaris*)^27^. We found significant degrees of variability of electrical spiking characteristics and substantial complexity of the electrical communication events^28^.

There are several reasons why studying the electrical activity of fungi is important. First is the biological understanding. Investigating the electrical activity of fungi provides valuable insights into the fundamental biological processes of these organisms. Understanding how fungi generate and propagate electrical signals can shed light on their physiological functions, communication mechanisms, and interactions with their environment. Second reason likes in the emergent properties of fungi. Fungi are fascinating examples of complex, self-organising systems. Their collective behaviour, emerging from the interactions of individual fungal cells, leads to intriguing phenomena such as spiking activity. By studying their electrical signals, we can explore the emergent properties of fungal colonies and mycelium networks, which have implications in various fields, including biophysics, bio-engineering and bio-computing. Third reasons lies in the environmental and agricultural implications. Fungi play crucial roles in ecosystems as decomposers, symbiotic partners in mycorrhizal associations, and, sometimes, as plant pathogens. By studying their electrical activity, we may gain insights into their ecological functions and how they interact with other organisms in their habitat. This knowledge can contribute to better agricultural practices, disease management, and ecological preservation. Fourth, and subjectively most important, reason is in the neuroscience connections. Fungal networks share similarities with neural networks found in animals and humans. By studying fungal electrical activity, we can explore analogies between fungal behaviours and neural processes, providing alternative perspectives and experimental models in the field of neuroscience.

In the experiments mentioned above we inserted electrodes in a substrate colonised by fungi. Topologies of the mycelium networks inside the colonised substrates have not been disclosed which complicated the interpretation of spiking activity. Therefore, we decided to conduct experiments on more homogeneous fungal material: colonies cultured on agar in Petri dishes. Results of this study are reported below.

## Methods

**Figure 1:**
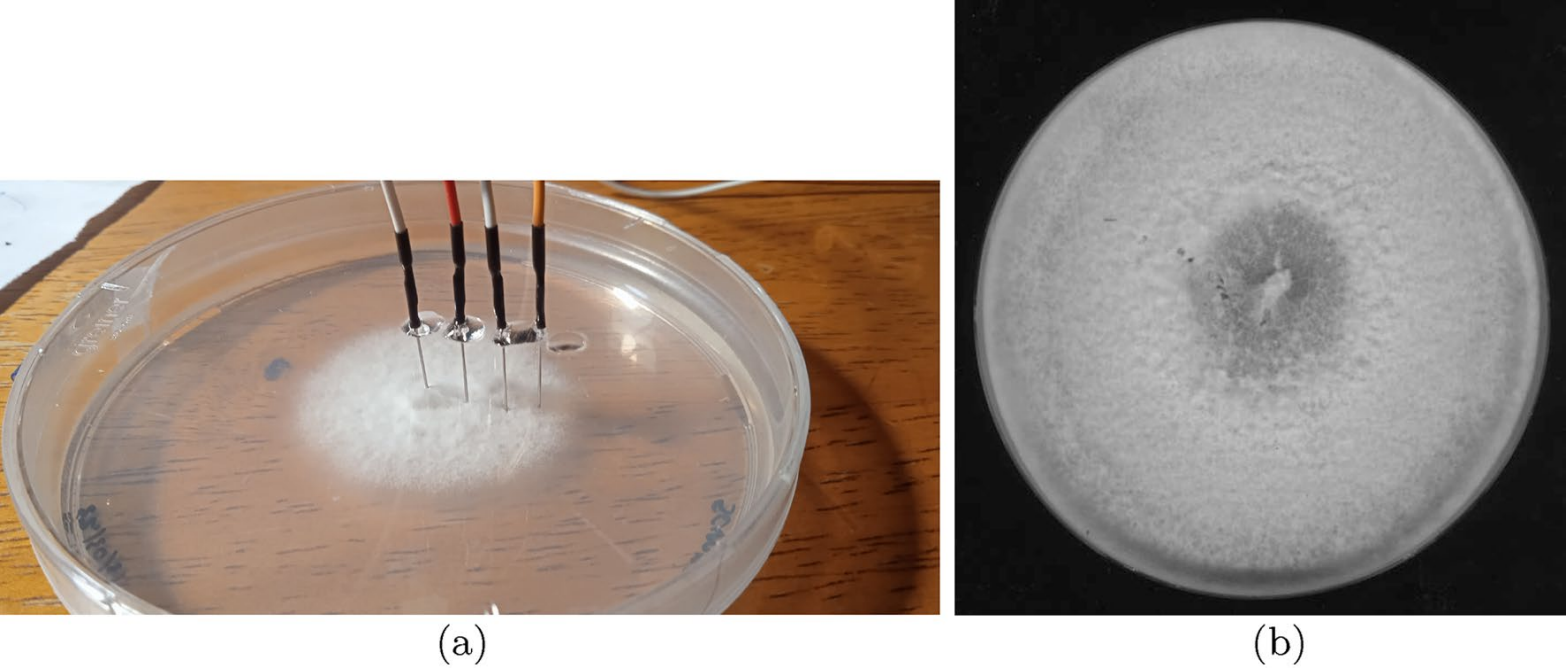
Experimental setup of recording electrical activity of the colony of *S. commune*. (**a**) Pairs of electrodes are positioned in contact with agar gel through the openings in the Petri dish’s lid and fixed with a glue. (**b**) Scan of the colony after ten days of the recording.

Split-gill fungus *Schizophyllum commune*, strain H4-8A (Utrecht University, The Netherlands) was grown on *S. commune* minimal medium (SCMM)^29^ supplemented with 1.5% agar for three days at $30\,^\circ$C in the dark. Petri dishes with fungal colonies were not opened during experiments. We melted openings in the Petri dish lids with a hot needle and inserted electrodes until they touched the bottom of the dishes (Fig. 1a). Three Petri dishes have been used. We started recordings c. 12–24 h after insertion of the electrodes. Electrical activity of the fungal colonies was recorded using pairs of iridium-coated stainless steel sub-dermal needle electrodes (Spes Medica S.r.l., Italy), with twisted cables and ADC-24 (Pico Technology, UK) high-resolution data logger with a 24-bit A/D converter. Galvanic isolation and software-selectable sample rates all contribute to a superior noise-free resolution. Each pair of electrodes reported a potential difference between the electrodes. In each pair of differential electrodes, the distance between electrodes was c. 10 mm. We recorded electrical activity at one sample per second. During the recording, the logger has been doing as many measurements as possible (typically up to 600 per s) and saving the average value. The acquisition voltage range was 78 mV. The recording continued for nearly 6 days. We have collected data from 16 pairs of differential electrodes. Due to inert coating the electrodes did not interfere with growth of the colonies as illustrated in Fig. 1b. The methodology of electrical recording was initially developed by us in 2018^25^ and well tested over the years on different species of fungi^26,27^.

**Figure 2:**
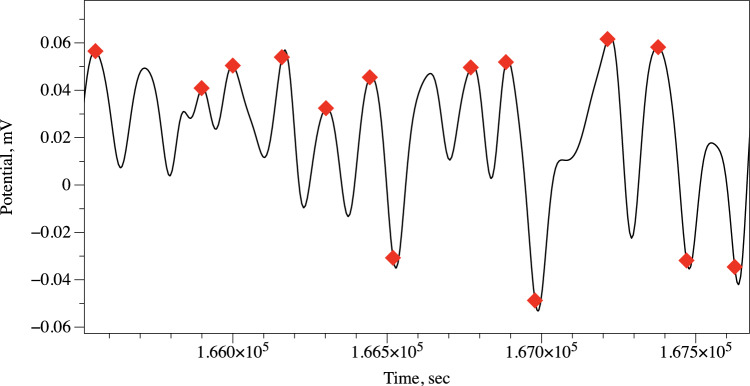
Example of spike detection.

Spiking activity of very slow and slow spikes (see definitions below) has be analysed manually due to low number of spikes. Fast spikes of electrical potential have been detected in a semi-automatic mode as follows; for each sample measurement $x_i$ we calculated the average value of its neighbourhood as $a_i=(4 \cdot w)^{-1} \cdot \sum _{i-2\cdot w \le j \le i+2\cdot w} x_j$. The index *i* is considered a peak of the local spike if $|x_i|-|a_i|>\delta$. The list of spikes were further filtered by removing false spikes located at a distance *d* from a given spike. Parameters specific to *S. commune* are $w=20$, $\delta =0.01$, $d=30$. An example of spike detection is shown in Fig. 2. The detection is not 100% (as any other algorithms) but over 90% having been detected, as per manual checks.

To illustrate a nature of electrical potential spikes we used FitzHugh–Nagumo (FHN) equations^30–32^. FHN model is which are a qualitative approximation of the Hodgkin–Huxley model^33^ of electrical activity of living cells:

1 $$\begin{aligned} \frac{\partial v}{\partial t}= & {} c_1 u (u-a) (1-u) - c_2 u v + I + D_u \nabla ^2, \end{aligned}$$

2 $$\begin{aligned} \frac{\partial v}{\partial t}= & {} b (u - v), \end{aligned}$$

where *u* is a value of a trans-membrane potential, *v* a variable accountable for a total slow ionic current, or a recovery variable responsible for a slow negative feedback, *I* is a value of an external stimulation current. We integrated the system using the Euler method with the five-node Laplace operator, a time step $\Delta t=0.015$ and a grid point spacing $\Delta x = 2$, while other parameters were $D_u=1$, $a=0.13$, $b=0.013$, $c_1=0.26$. We controlled excitability of the medium by varying $c_2$ from 0.05 (fully excitable) to 0.015 (non excitable). Boundaries are considered to be impermeable: $\partial u/\partial {\textbf{n}}=0$, where ${\textbf{n}}$ is a vector normal to the boundary. We simulated electrodes by calculating a potential $p^t_x$ at an electrode location *x* as $p_x = \sum _{y: |x-y|<2} (u_x - v_x)$. The simulation was conducted on a grid $300 \times 300$ nodes. Source of the one-off excitation was placed in the middle of the grid, coordinates (150, 150). Active electrode *x* was placed at coordinates (190, 150) and reference electrode *y* at coordinates (190+d, 150), where $d=1, 5, 10, 40, 80$.

## Results

**Figure 3:**
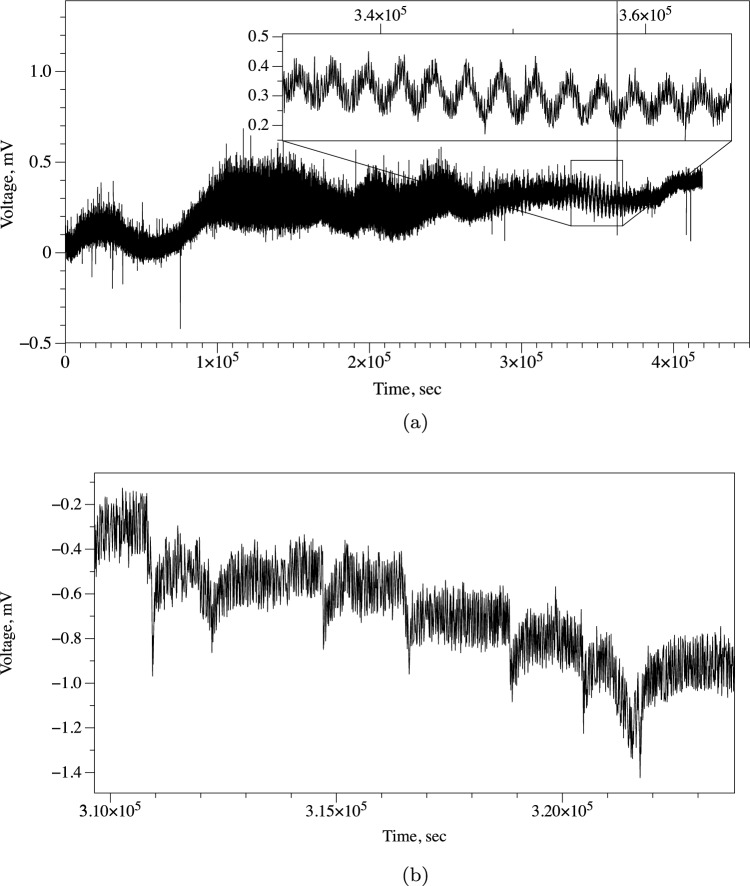
Emergence of regular electrical activity in cultures of *S. commune*. Slow and very slow spikes are shown. (**a**) Train of symmetrical spikes is magnified in the insert. (**b**) Action potential-like spikes.

Typically, recordings obtained in experiments consist for 80% of duration of irregular patterns of wide band electrical activity with no pronounced regular patterns. However, there are segments of activity, where trains of regular spikes of electrical potential are presented. This is illustrated in experimental recording plotted in Fig. 3, where a train of spikes is shown in the magnified insert.

Manual analysis of the recordings established three scales of electrical potential oscillations: (1) very slow spikes, an hour range, (2) fast spikes, few minutes range, and (3) very fast spikes, less than half a minute range.

### Very slow spikes

In trains of symmetrical spikes, an average duration of a spike is 2573 s (median 2630 s, $\sigma$ = 168) and an average amplitude is 0.16 mV (median 0.16 mV, $\sigma$ = 0.02). An average distance between spikes is 2656 s (median 2630 s, $\sigma$ = 278). Each spike is symmetrical, i.e. a temporal distance from the start of the spike to the summit is equal to a temporal distance from the summit to the end of the spike.

### Slow spikes

A train of action potential-like spikes is illustrated in Fig. 3b. An average duration of a spike is 457 s (median 424 s, $\sigma$ = 120), average amplitude is 0.4 mV (median 0.4 mV, $\sigma$ = 0.10). An average distance between spikes is 1819 s (median 1738 s, $\sigma$ = 532). The spikes are temporally asymmetric: average duration from start of a spike to its summit is 89 s (media 83 s, $\sigma$ = 59). That is 20% of the average width of a spike.

**Figure 4:**
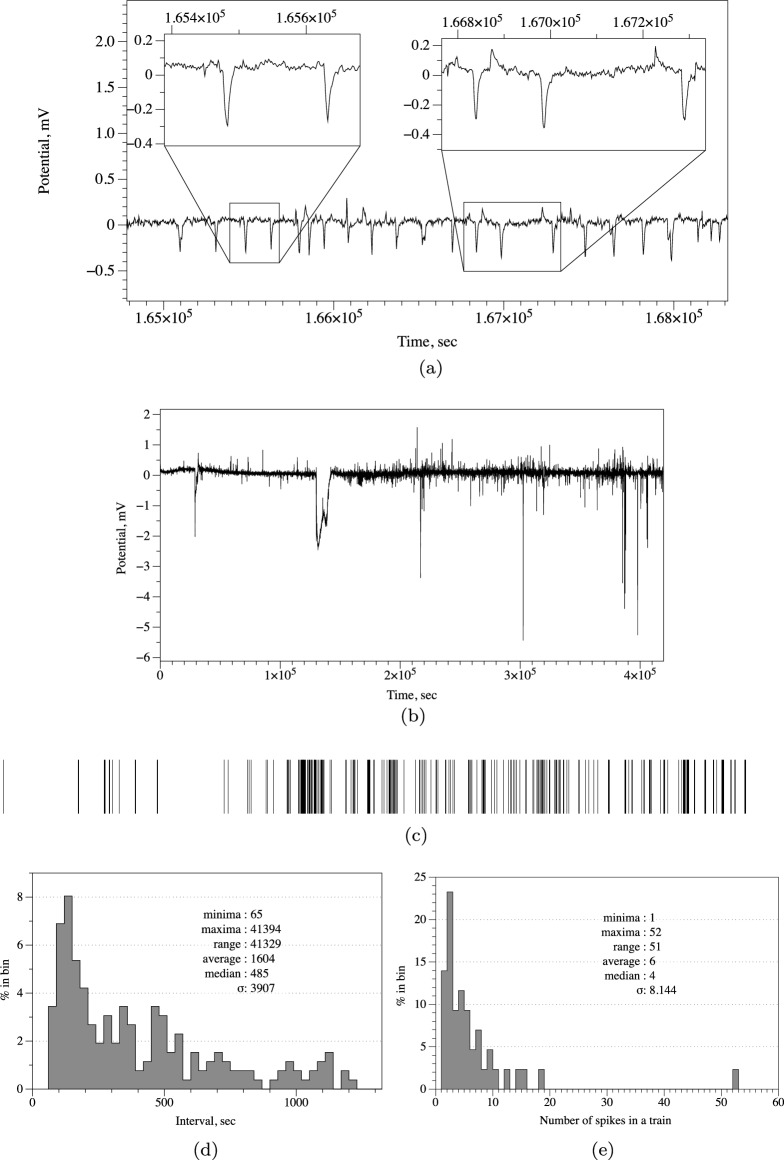
Fast action potential-like spikes in cultures of *S. commune*. (**a**) Example of a train of spikes with most characteristic spikes zoomed in the insert. (**b**) Recording conducted during 5 days. (**c**) Spikes detected represented as bar code. (**d**) Distribution of distances between fast spikes. (**e**) Distribution of a number of spikes in a train of spikes.

### Very fast spikes

Third family of action potential-like spikes discovered are fast spikes (Fig. 4a). Average width of fast spikes is 24 s (median 23 s, $\sigma$ = 0.07). Average amplitude is 0.36 mV (media 0.35 s, $\sigma$ = 0.06). In the fragment illustrated in Fig. 4a, an average distance between spikes is 148 s (media 143 s, $\sigma$ = 68). However, in the overall recording of 5 days (Fig. 4b), the characteristics of the spikes vary substantially. A bar-code representation of the spikes is shown in Fig. 4c. Distance between spikes varies from 65 s to 114 min (Fig. 4d). The spikes do appear in trains, an average train length is six spikes and median four spikes, $\sigma$ = 8.1 (Fig. 4e).

**Figure 5:**
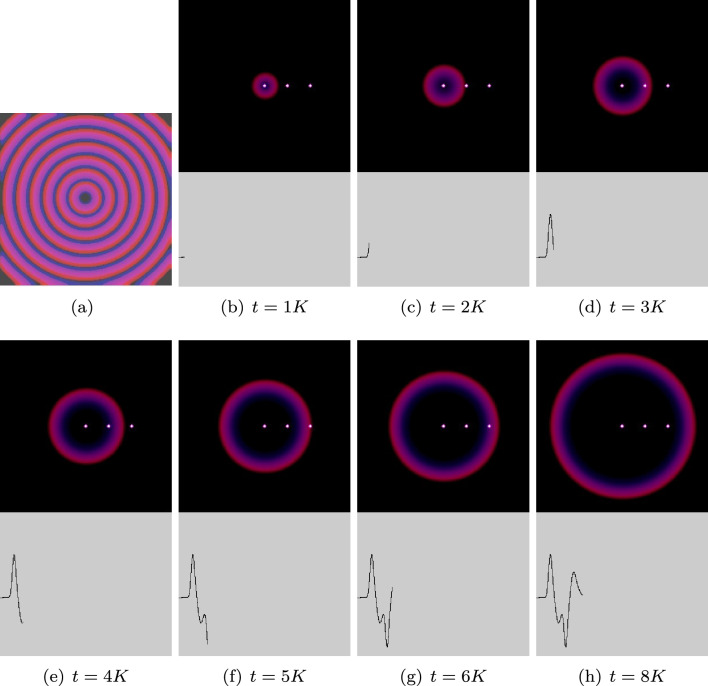
Modelling spiking activity. (**a**) Propagation of excitation wave in a conducive space imitating colony of *S. commune*. The image is an overlap of time lapse snapshots of a single wave, saved every 1500th iteration. (**b–j**) Snapshots of excitation dynamics recorded at 1K iterations of numerical integration of FHN model to 9K iterations. Left white dot is a source of excitation. Two right white dots is a pair of differential electrodes. A potential difference between the electrodes is shown at the bottom of each snapshot. Distance between electrodes is 40 nodes of the integration grid.

**Figure 6:**
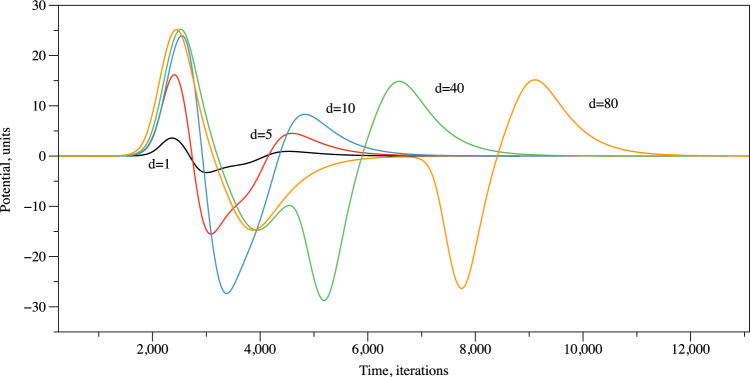
Spikes recorded in simulation with various distance between electrodes.

To demonstrate potential mechanisms of spiking we used Fitz–HughNagumo (FHN) model. We represented a source of electrical excitation at the centre of the colony. Due to the colony being nearly homogeneous—at the macro scale—the wave of excitation propagates as a circular wave (Fig. 5a). When a wave of excitation crosses a loci between the electrodes, a potential difference is recorded. This is illustrated in Fig. 5. In this example, the left electrode is active and the right electrode is a reference. A potential is zero when an excitation wave is far from the electrodes (Fig. 5b). As soon as the wave front starts propagating under the active electrode a positive electrical potential is recorded (Fig. 5c,d). When the wave front approaches the reference electrodes, the value of the electrical potential recorded on the active electrode drops to below zero (Fig. 5e,f). When a negatively charged tail of the wave front passes under the reference electrode, a second peak of positive electrical potential is recorded on the active electrode (Fig. 5g,h). In Fig. 6, we illustrate how the distance *d* between electrodes might affect the shape of the spike recorded. A spike has two extremes when $d=1$, three extremes for $d=5, 10$, and five extremes when $d=40, 80$. Indeed, we should keep in mind that an excitation front width in the model was c. 15 nodes.

## Discussion

We observed three families of regular electrical activity in colonies of *S. commune*. We observed very slow spikes with a duration of c. 43 min, slow spikes with a duration of c. 8 min and very fast spikes of a c. 24 s duration, on average. Thus, the three temporal scales of spiking represent three types of waves, or propagating patterns, which causes changes in the electrical potential. What could be a reason for such a high degree of variability in the speed of propagating excitation? A translocation of metabolites could be the most realistic answer. A study on continuous imaging of amino-acid translocation in mycelia of *Phanerochaete velutina*^34^ uncovered pulsating fluxes in which the speed of propagation varies from 9 to 51 mm/h. In our experiments, a distance between electrodes in each differential pair was about 10 mm. This means that c. 9 mm/h translocation speed, reported in Ref.^34^, matches the very slow spikes of electrical potential that were measured. The highest speed of translocation, 51 mm/h, roughly matches the slow spikes with an average duration of 8 min. Moreover, slow spikes with 8 min duration can be also be related to propagation of calcium waves. Assuming the fastest calcium wave, as per review^35^, propagates at 0.03 mm/s, the wave will travel between electrodes in c. 5 min. Very fast spikes, indicating a speed of excitation propagation of c. 1500 mm/h are unlikely to be related to transport of metabolites. However, there is a sufficient amount of experimental evidence, see an overview in Refs.^36,37^, that fungi grow in a pulsating manner rather than at a constant rate. Namely, *Neurospora crassa* shows 3–6 s pulse, and a speed of 201 nm/s, while *Trichoderma virde* 4–6 s pulse and 201 nm/s speed^36^. In seven species of fungi studied in Ref.^36^, pulsing varies from 4 to 30 s in duration. We can therefore hypothesise that fast action potential spiking is associated with, or even controls, a pulsating growth of *S. commune*.

Compared to our prior research on recording the electrical activity of fungi in colonized substrates^25–27^, we have verified that the electrical signals captured from fungal colonies on agar gel exhibit a higher signal-to-noise ratio and more pronounced spike shapes. These signals can be likened to the electrical spiking activity observed in a pure mycelium grown on a liquid culture, referred to as “fungal skin” in our recent study.

The wave of electrical activity propagating in the fungal colonies, might also be employed in sensorial fusion, information transfer, and distributed decision making. We will explore this possibility in our future research.

## Data Availability

The datasets used and/or analysed during the current study available from the corresponding author on reasonable request.
